# Treatment experience in managing severe immune-mediated hepatotoxicity induced by immune checkpoint inhibitors

**DOI:** 10.3389/fonc.2025.1657332

**Published:** 2025-10-10

**Authors:** Ruijie Cao, Shasha Zhang, Jingjing Zhang, Yufei Zhao, Xiaoyun Zhang, Zhanjun Guo

**Affiliations:** Department of Immunology and Rheumatology, The Fourth Hospital of Hebei Medical University, Shijiazhuang, China

**Keywords:** immune-mediated hepatotoxicity, immune checkpoint inhibitors, immune-related adverse events, steroid, plasma exchange

## Abstract

**Background:**

Although multiple guidelines for managing severe immune-mediated hepatotoxicity (IMH) induced by immune checkpoint inhibitors (ICIs) were recommended, real-world research data regarding its clinical progression, treatment modalities, and outcomes remain scarce.

**Methods:**

This study was a retrospective, single-center investigation conducted at the Fourth Hospital of Hebei Medical University. A total of 379 patients with complete clinical records were enrolled in the Department of Immunology and Rheumatology at the Fourth Hospital of Hebei Medical University from January 2021 to July 2024. Binary logistic regression analysis was employed to identify potential risk factors associated with the development of severe IMH or infection during treatment. Receiver operating characteristic (ROC) curve analysis was further applied to determine the optimal cutoff values for the identified risk factors.

**Results:**

A total of 32 severe IMH patients were analyzed for risk factor association; the Eastern Cooperative Oncology Group performance status (ECOG PS) (p = 0.001) and oral traditional Chinese medicine (TCM) (p < 0.001) were independently associated with the incidence of severe IMH. Based on our experience with these 32 patients, a road map was proposed for the management of severe IMH patients: conventional applications of daily methylprednisolone sodium succinate with dose adjustment for 11 days, a liver biopsy to exclude vanishing bile duct syndrome for steroid-resistant patients, and subsequent plasma exchange (PE). Furthermore, cumulative steroid use was identified as an independent risk factor for concurrent infection with a cutoff value of 1,656 mg (p = 0.024) in severe IMH patients.

**Conclusion:**

We investigated the risk factor of severe IMH and provided a feasible treatment roadmap for severe IMH.

## Introduction

Immune checkpoint inhibitors (ICIs) have revolutionized the therapeutic landscape for advanced cancer patients, including melanoma, non-small cell lung cancer, esophageal cancer, gastric cancer, and hepatocellular carcinoma (1–6). ICIs can enhance the activity of immune cells and promote the immune system’s attack on tumor cells by inhibiting the immune checkpoint molecules such as cytotoxic T-lymphocyte-associated protein 4 (CTLA-4), programmed death 1 (PD-1), and programmed cell death ligand 1 (PD-L1). The activation of the immune system upon ICI treatment may initiate the inflammatory side effects, which are defined as immune-related adverse events (irAEs) (7) with a higher incidence in patients receiving CTLA-4 (53.8%) treatment when compared with those receiving ICIs targeting PD-1 (26.5%) or PD-L1 (17.1%) (8). IrAEs have the potential to target any organ system, with the most frequent toxicities for the integumentary, gastrointestinal, and endocrine systems. The manageable lower-grade irAEs with minimal treatment intervention of ICI treatment are associated with a favorable prognosis (9, 10), while higher-grade irAEs with potentially life-threatening risk may need permanent treatment discontinuation (11). The underlying mechanisms of irAEs are still being investigated. The potential mechanisms include increased T-cell activity, elevated levels of pre-existing autoantibodies, heightened inflammatory cytokine levels, and enhanced complement-mediated inflammation (7).

Liver-related irAEs are referred to as immune-mediated hepatotoxicity (IMH), with severe IMH defined as those meeting Common Terminology Criteria for Adverse Events (CTCAE) grade 3 or higher (12, 13). Notably, severe IMH occurs in approximately 1%–2% of patients undergoing anti-PD-1 or anti-PD-L1 therapy, whereas the incidence can increase to as high as 10% in patients receiving combination therapy with anti-CTLA-4 and anti-PD-1 agents (14). The patients with pre-existing liver injury, such as viral hepatitis B, metabolic disorders, and liver metastases, are more prone to IMH incidence (15). The mechanisms by which hepatocytes are targeted in IMH remain incompletely understood. However, recent studies have shown that liver tissue from patients with IMH exhibits a pattern of immune cell infiltration similar to that seen in peripheral tissues of patients treated with ICIs. This infiltration is characterized by the accumulation of CD8+ T cells and CCR2+ macrophages (16). Therefore, the extensive infiltration and excessive activation of lymphocytes upon ICI treatment may represent a critical underlying mechanism contributing to the development of IMH (17).

The multiple guidelines for managing IMH from the American Society of Clinical Oncology (ASCO) (14) and the National Comprehensive Cancer Network (NCCN) (18) were recommended, but all current recommendations are mainly based on expert consensus rather than evidence-based medicine (19). There remains a notable lack of real-world studies to evaluate the risk factors, clinical progression, and particularly the treatment experience [the optimal timing of steroid administration, the assessment of steroid resistance, the timing and practical value of liver biopsy, and the appropriate time point for plasma exchange (PE)] (20). While several small-sample studies have investigated the risk factors for severe IMH, the results are not consistent regarding clinical application (17–19). To identify the risk factors associated with the development of severe IMH and to evaluate the treatment decisions for this condition, we conducted a retrospective analysis of cancer patients who developed severe IMH following ICI treatment.

## Methods

### Study design and population

The study included a total of 379 patients with complete clinical records admitted to the Department of Immunology and Rheumatology at the Fourth Hospital of Hebei Medical University from January 2021 to July 2024. The inclusion criteria were as follows: a) confirmed diagnosis of solid tumors based on pathological examination, b) a history of immunotherapy defined as having received at least one cycle of immunotherapy, and c) availability of complete clinical data. The exclusion criteria were as follows: a) patients with alternative causes of abnormal hepatic function aside from ICIs, including active viral hepatitis, active autoimmune hepatitis, primary cholestatic disorders, biliary obstruction, circulatory disorders, sepsis, other drugs, or cancer progression; b) the presence of severe cardiopulmonary insufficiency or other significant systemic diseases; and c) total bilirubin (TBIL) did not reach CTCAE grade 3 in IMH patients.

IMH is defined as aspartate aminotransferase, or alanine aminotransferase, or TBIL elevation (according to CTCAE Version 5.0 specific cutoffs), ranging from grade 1 to grade 5 (13). Since the increase in transaminase levels does not present therapeutic challenges, severe IMH in this study was defined as TBIL elevation of grade 3 or higher, referring to hepatotoxicity. A standardized scoring system of the Roussel Uclaf Causality Assessment Method (RUCAM) (21) was utilized to assess the causal relationship between ICIs and IMH. Among these cancer patients, 32 individuals with TBIL levels ≥3 times the upper limit of normal met the criteria for severe IMH. Among these patients, 31 cases were transferred to our department for treatment following a confirmed diagnosis of severe IMH after receiving ICI therapy.

Baseline characteristics included gender, age, Eastern Cooperative Oncology Group performance status (ECOG PS), cancer type, the presence of liver metastasis, underlying liver disease etiology, and specific immunotherapy details (drug types, therapeutic protocols, and treatment cycle). The timing of onset and recovery of severe IMH, corticosteroid dosage administered during treatment, the application of immunosuppressive agents, PE therapy, and the occurrence of infection as a treatment-related complication were documented. Laboratory data encompassing peak levels of TBIL and monitoring changes in bilirubin levels throughout the course of treatment were also included.

### Statistical analysis

Data analysis was performed using IBM SPSS software version 26.0 (IBM Corp., Armonk, NY, USA). Categorical variables were expressed as frequencies and percentages, with group comparisons conducted using the chi-square test or Fisher’s exact test. Continuous variables were presented as mean±SD for normally distributed data, or as median with interquartile range (IQR) for non-normally distributed data. Between-group comparisons were carried out using either Student’s t-test or the Mann–Whitney U test, depending on the distributional characteristics of the data. The association between covariates and binary dependent variables was analyzed using a logistic regression model. The variables included in the multivariate analysis were selected based on a comprehensive consideration of indicators with statistical significance in univariate analysis and clinically relevant factors. Receiver operating characteristic (ROC) curve analysis was employed to determine the cutoff values for identifying risk factors. p-Values ≤0.05 were considered statistically significant.

## Results

### Clinical background of the patients

A total of 348 cancer patients who received immunotherapy in our department were screened, with one case fitting the severe IMH criteria, while the other 31 cases that transferred to our department for IMH treatment, following the diagnosis of severe IMH, were also included in the present analysis. The ICIs used in the present cohort consisted of 375 cases of anti-PD-1 antibodies (including sintilimab, camrelizumab, tislelizumab, pembrolizumab, toripalimab, and serplulimab) and four cases of anti-PD-L1 antibody therapy (adebrelimab). The incidence of severe IMH was the highest in lung cancer patients, with nine cases occurring out of 45 patients (20%), followed by esophageal cancer patients at 17.4% (12 out of 69 patients) and gastric cancer at 5.3% (10 out of 189 patients). The median age for the total patients was 65 years (IQR, 57, 71 years), comprising 272 men and 107 women, but the median age of severe IMH patients was 69 years (IQR, 59, 73 years), comprising 25 men and seven women. Among these patients, 15 patients received ICI monotherapy, whereas 364 patients received ICI therapy combined with chemotherapy or targeted therapy. Some patients presented with basic liver diseases, with 53 cases of liver metastasis and 31 cases of concurrent hepatitis B virus infection at the initiation of ICI therapy, but all patients displayed preserved hepatic function (**Table 1**).

**Table 1:** The baseline characteristics of patients with severe IMH and the overall population at the initiation of ICI therapy.

	IMH No. (%)	Total No. (%)
Age (years)		
≤65	12 (37.5)	198 (52.2)
>65	20 (62.5)	181 (47.8)
Gender		
Male	25 (78.1)	272 (71.8)
Female	7 (21.9)	107 (28.2)
Primary cancer		
Lung cancer	9 (28.1)	45 (11.9)
Esophageal cancer	12 (37.5)	69 (18.2)
Gastric cancer	10 (31.3)	189 (49.9)
Malignant melanoma	1 (3.1)	8 (2.1)
Colorectal cancer	0 (0.0)	8 (2.1)
Primary liver cancer	0 (0.0)	29 (7.7)
Hypopharyngeal cancer	0 (0.0)	11 (2.9)
Laryngeal cancer	0 (0.0)	7 (1.8)
Renal cancer	0 (0.0)	5 (1.3)
Urothelial cancer	0 (0.0)	4 (1.1)
Thyroid cancer	0 (0.0)	3 (0.8)
Parotid gland cancer	0 (0.0)	1 (0.3)
ICIs		
Sintilimab	8 (25.0)	79 (20.8)
Camrelizumab	12 (37.5)	94 (24.8)
Tislelizumab	11 (34.4)	126 (33.2)
Adebrelimab	1 (3.1)	4 (1.1)
Pembrolizumab	1 (3.1)	58 (15.3)
Toripalimab	0 (0.0)	6 (1.6)
Serplulimab	0 (0.0)	12 (3.2)
Immunotherapy regimens		
Monotherapy	2 (6.3)	15 (4.0)
Combined chemotherapy	28 (87.5)	238 (62.8)
Combined targeted therapy	1 (3.1)	74 (19.5)
Combined chemotherapy and targeted therapy	1 (3.1)	52 (13.7)
Hepatic metastases		
N	31 (96.9)	326 (86.0)
Y	1 (3.1)	53 (14.0)
Infection with hepatitis B virus		
N	29 (90.6)	348 (91.8)
Y	3 (9.4)	31 (8.2)
ECOG PS		
≤1	26 (81.3)	185 (48.8)
>1	6 (18.8)	194 (51.2)

IMH, immune-mediated hepatotoxicity; ICIs, immune checkpoint inhibitors; ECOG PS, Eastern Cooperative Oncology Group performance status; N, no; Y, yes.

### Clinical features of severe IMH

The clinical characteristics of 32 severe IMH patients, with 12 patients of grade 3 and 20 patients of grade 4 severity, according to CTCAE, are presented in **Table 2**. The onset of IMH occurred at a median of 71 days (IQR, 45, 128 days) following the initiation of ICI therapy. The average value of the highest TBIL level was 283±108 um/L. Additionally, 18 patients reported concurrent oral use of traditional Chinese medicine (TCM) (including cinobufacin, Xihuang pill, and *Marsdenia tenacissima*) during their ICI treatment.

**Table 2:** Clinical features of severe IMH. .

Grade of IMH (3/4)	12/20
The maximum level of TBIL (μmol/L)	283±108
Oral adjuvant anti-tumor TCM (y/n)	18/14
The dose of steroid = 2 mg·kg^−1^·day^−1^ during treatment (y/n)	16/16
Duration of steroid (days)	44±22
Cumulative dosage of steroid (mg)	1,240 (899, 1,852)
Mycophenolate mofetil was used (y/n)	7/25
Tocilizumab was used (y/n)	2/30
Underwent plasma exchange (y/n)	8/24
Infection (y/n)	6/26
Time to onset of IMH (days)	71 (45, 128)
Time to onset of improvement with steroid therapy (days)	4 (3, 11)
Time to resolution of IMH (days)	66 (52, 79)

IMH, immune-mediated hepatotoxicity; TBIL, total bilirubin; TCM, traditional Chinese medicine; y, yes; n, no.

### Analysis of risk factors for severe IMH

To investigate the risk factors contributing to the development of severe IMH, an assessment of patient background factors at the initiation of ICI therapy was conducted. Univariate analysis indicated that the type of cancer [lung cancer (p = 0.007), esophageal cancer (p = 0.003), and gastric cancer (p = 0.028)], pembrolizumab application (p = 0.046), ECOG PS (p < 0.001), and oral TCM (p < 0.001) were significantly associated with the incidence of severe IMH (**Table 3**). A multivariate binary logistic regression analysis indicated that ECOG PS [odds ratio (OR) 0.191; 95% confidence interval (CI) 0.075–0.487; p = 0.001] and oral TCM (OR 4.781; 95% CI 2.207–10.355; p < 0.001) were independently associated with severe IMH (**Table 4**).

**Table 3:** Factors associated with the incidence of severe IMH (univariate analysis).

	IMH (n = 32)	Non-liver injury (n = 347)	χ^2^/z	P-value
Age (≤65/>65)	12/20	186/161	3.045	0.081
Gender (M/F)	25/7	247/100	0.697	0.404
Primary cancer				
Lung cancer (y/n)	9/23	36/311	7.207	0.007
Esophageal cancer (y/n)	12/20	57/290	8.737	0.003
Gastric cancer (y/n)	10/22	179/168	4.846	0.028
Primary liver cancer (y/n)	0/32	29/318	1.834	0.176
Others (y/n)	1/31	46/301	1.914	0.166
ICIs				
Sintilimab (y/n)	8/24	71/276	0.366	0.545
Camrelizumab (y/n)	12/20	82/265	3.022	0.082
Tislelizumab (y/n)	11/21	115/232	0.020	0.887
Adebrelimab (y/n)	1/31	3/344		0.298
Pembrolizumab (y/n)	1/31	57/290	4.000	0.046
Others (y/n)	0/32	18/329	0.785	0.376
HBsAg (+/−)	3/29	28/319	<0.001	1.000
Hepatic metastases (y/n)	1/31	57/290	3.039	0.081
Immunotherapy cycle (>3/≤3)	13/19	188/159	2.161	0.142
Immunotherapy regimens (monotherapy/combination)	2/30	13/334	0.049	0.825
Oral TCM (y/n)	18/14	76/271	18.533	<0.001
ECOG PS (>1/≤1)	6/26	188/159	14.718	<0.001

IMH, immune-mediated hepatotoxicity; M, male; F, female; y, yes; n, no; ICIs, immune checkpoint inhibitors; HBsAg, hepatitis B surface antigen; TCM, traditional Chinese medicine; ECOG PS, Eastern Cooperative Oncology Group performance status.

**Table 4:** Factors associated with the incidence of severe IMH (multivariate analysis). .

	OR	95% CI	P-value
Oral TCM	4.781	2.207–10.355	<0.001
ECOG PS > 1	0.191	0.075–0.487	0.001
Pembrolizumab	0.169	0.022–1.308	0.089

IMH, immune-mediated hepatotoxicity; OR, odds ratio; CI, confidence interval; TCM, traditional Chinese medicine; ECOG PS, Eastern Cooperative Oncology Group performance status.

### Treatment of severe IMH

The median total dose of methylprednisolone sodium succinate administered in the present study was 1,240 (IQR, 899, 1,852) mg, with an average duration of steroid use lasting for approximately 44±22 days. The median time to resolution of severe IMH was 66 (IQR, 52, 79) days (**Table 2**). The dynamic changes in TBIL levels upon steroid treatment are presented in **Supplementary Figure S1**. In the present study, methylprednisolone sodium succinate was typically administered at a daily dose of almost 1 mg/kg with liver function monitored every 3 days. The median time for a reduction of TBIL levels among the 32 patients was observed on day 4 after the commencement of steroid therapy. For the remaining patients whose TBIL levels did not decrease by day 4 upon steroid treatment, the increased daily dosage of 2 mg/kg, followed by dose adjustment along with TBIL changes, also resulted in TBIL reduction in approximately 8 (50%) patients at day 11. The prolonged steroid usage did not decrease the TBIL level after that time point; therefore, steroid resistance was defined at that time point. In the present study, two or three sessions of PE therapy were administered to a total of eight patients, including seven patients who had shown steroid-refractory responses and one patient who had not yet received steroid treatment. Notably, the patient who received PE prior to steroid administration experienced a delay in the reduction of TBIL, resulting in an escalated steroid dosage and complications associated with infection. Three out of the seven patients with steroid-refractory conditions did not exhibit a decrease in TBIL levels following PE and eventually died. One of these patients achieved bilirubin normalization following intensive therapy and resumed ICI treatment 1 year later due to oncologic treatment requirements. However, this was followed by a severe recurrence of IMH, which ultimately resulted in the patient’s death. Additionally, one of the three patients underwent liver biopsy, which confirmed the presence of vanishing bile duct syndrome. The remaining two patients received tocilizumab treatment after PE, which resulted in a modest reduction in TBIL levels; unfortunately, these two patients eventually succumbed to *Pneumocystis carinii* (P.c.) infection and intestinal perforation.

### Analysis of risk factors for concurrent infection after steroid therapy

**Figure 1:**
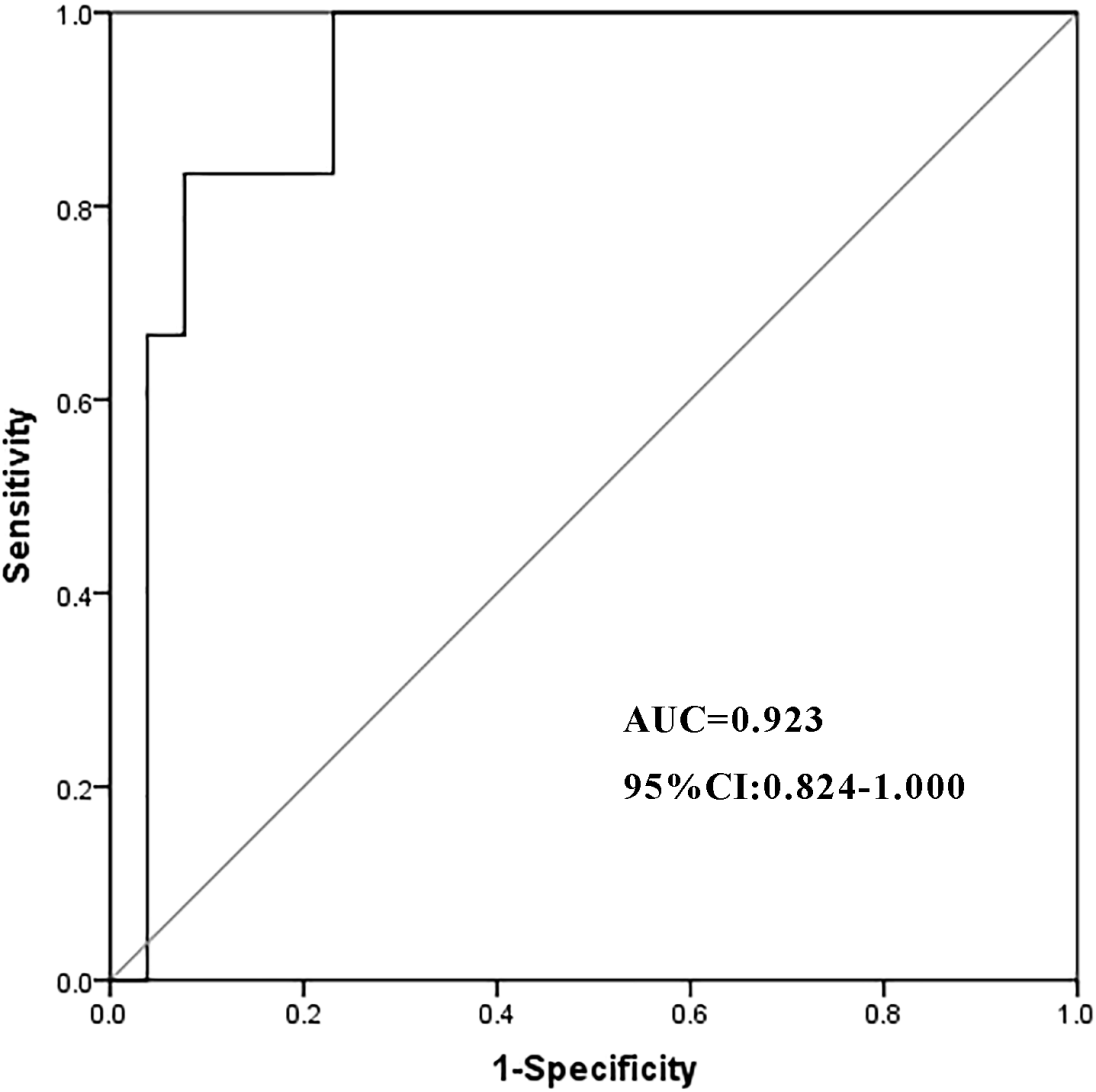
The ROC curve of the cumulative dosage of methylprednisolone. ROC, receiver operating characteristic; AUC, area under the curve; CI, confidence interval.

In a cohort of 32 patients with severe IMH following steroid therapy, six individuals developed infections, including two cases of viral infection, three cases of P.c. infection, and one case of bacterial pneumonia. Univariate analysis of clinical characteristics revealed that higher cumulative doses of steroids (p = 0.001) and elevated TBIL levels (p = 0.039) were significantly associated with the risk of infection after steroid therapy (**Table 5**). The cumulative dose of methylprednisolone (OR 1.001; 95% CI 1.000–1.003; p = 0.024) was further identified as an independent risk factor for infection in a multivariate binary logistic regression analysis (**Table 6**). ROC curve analysis was performed to evaluate the predictive capacity of the cumulative methylprednisolone dosage for infection, which revealed an area under the curve (AUC) of 0.923. The analysis also identified a cutoff value of 1,656 mg as a threshold for predicting the infection risk (**Figure 1**).

**Table 5:** Factors associated with the occurrence of infection (univariate analysis).

	No-infection	Infection	P-value
Age (≤65/>65)	10/16	2/4	1.000
Gender (M/F)	20/6	5/1	1.000
ECOG PS (>1/≤1)	5/21	1/5	1.000
Primary cancer (lung cancer/esophageal cancer/gastric cancer/others)	5/10/10/1	4/2/0/0	0.103
HBsAg (+/−)	2/24	1/5	0.476
Hepatic metastases (y/n)	1/25	0/6	1.000
Immunotherapy cycle (>3/≤3)	9/17	4/2	0.194
Grade of IMH (3/4)	12/14	0/6	0.061
Immunotherapy regimens (monotherapy/combination)	2/24	0/6	1.000
Mycophenolate mofetil was used (y/n)	5/21	2/4	0.590
Tocilizumab was used (y/n)	1/25	1/5	0.345
Cumulative dosage of steroid (mg)	1,110 (839, 1,630)	3,112 (1,873, 3,553)	0.001
Duration of steroid administration (days)	42±23	50±13	0.440
The maximum level of TBIL(μmol/L)	264±109	354±55	0.039

M, male; F, female; ECOG PS, Eastern Cooperative Oncology Group performance status; HBsAg, hepatitis B surface antigen; y, yes; n, no; IMH, immune-mediated hepatotoxicity; TBIL, total bilirubin.

**Table 6:** Factors associated with the independent risk factors of infection (multivariate analysis).

	OR	95% CI	P-value
Cumulative dosage of steroid (mg)	1.001	1.000–1.003	0.024
The maximum level of TBIL (μmol/L)	1.004	0.990–1.019	0.565

OR, odds ratio; CI, confidence interval; TBIL, total bilirubin.

## Discussion

To the best of our knowledge, the present study is the largest single-center cohort investigation to date that examined the clinical characteristics of patients who developed severe cholestatic IMH, including their clinical course, treatment methods, and outcomes.

The reported incidence of IMH varies significantly, ranging from 0.7% to 16%. This variation depends on factors such as the specific class of ICIs, dosage regimen, and whether it is monotherapy or combination therapy with ICIs (22). Approximately 1%–2% of patients receiving anti-PD-1/anti-PD-L1 therapy will develop grade 3 or higher IMH (23). Among these 32 patients, only one case developed severe IMH following immunotherapy provided by our center. The rest of the patients were transferred to our center after being diagnosed with IMH elsewhere. Therefore, the incidence in our cohort (1/348) was comparable to that reported in other studies. In the univariate analysis of risk factors for severe IMH, cancer type, specifically lung, esophageal, and gastric cancers, was found to be associated with the incidence of severe IMH. However, given that the majority of severe IMH patients in our center were referred from the thoracic surgery department, a significant selection bias was introduced regarding the types of cancer. Consequently, these variables were not included in the multivariate analysis of severe IMH risk factors.

The pathophysiology of IMH is not fully understood. The liver injury observed in IMH seems to be primarily related to the inherent mechanism of action of ICIs. Specifically, it is due to the increased autoimmunity to hepatocytes resulting from ICI-induced T-cell activation, rather than their intrinsic hepatotoxicity or immunogenicity, which is typically seen in other types of drug-induced liver injury (DILI) (24). In the present study, 56.3% of patients with severe IMH received oral TCM such as cinobufacin, Xihuang pill, and *M. tenacissima* as a part of their cancer treatment regimen. TCM is a major contributor to DILI among Asian populations; however, its underlying mechanisms remain incompletely understood (25). The pathogenesis of herb-induced liver injury may involve oxidative stress, inflammatory responses, mitochondrial dysfunction, cell apoptosis, lipid metabolism, and the release of fibrosis-promoting factors (26). We employed the RUCAM scoring system to diagnose IMH in order to exclude potential interference form TCM. However, due to the complexity of TCM components and the variability in administration methods, it remains challenging to fully eliminate the influence of DILI. Our findings suggest that TCM use may represent an independent risk factor for severe IMH. Although the inclusion of transferred patients from other departments or hospitals, as well as the potential hepatotoxic effects of TCM, may introduce some statistical bias in the analysis of the overall patient population, these findings still strongly remind us to exercise greater caution in the application of TCM during ICI treatment. Furthermore, a lower ECOG PS score ≤1 was identified as a potential risk factor for cholestatic IMH, possibly attributed to the inclination of patients with better functional status to receive higher doses of combination therapy, including chemotherapy and targeted therapy. In the present study, liver function showed signs of improvement at a median of 4 days. Half of the patients with an initially poor response to steroids displayed improvement in liver function after continued steroid use with increased doses at day 11. Beyond this time point, continued steroid therapy does not lead to improvement of liver function; therefore, we defined steroid resistance for IMH treatment as no improvement in liver function after 11 consecutive days of methylprednisolone treatment with the conventional dose of 1–2 mg·kg^−1^·day^−1^. The NCCN guidelines for managing IMH recommend adding mycophenolate mofetil (MMF) after 3–5 days of steroid therapy if the response is inadequate (18), but based on our clinical experience, it occupies only a very subordinate auxiliary position in managing liver function. In our study, seven patients who showed poor responses to steroid therapy were treated with MMF; however, none of them exhibited a significant decrease in bilirubin levels. Among these seven patients, four only experienced a notable decline in bilirubin levels following PE therapy. Based on this, the potentially most feasible solution may be PE. The necessity of liver biopsy in severe IMH patients is controversial (27), but we found an incurable bile duct deficiency patient among the steroid-refractory patients. Therefore, we recommend liver biopsy after confirmed steroid resistance but before PE to avoid unnecessary expenses for the bile duct deficiency patient.

PE has been utilized in the management of severe cholestatic liver injury (28, 29), including liver irAEs (30). It can clear the pathological mechanisms of irAEs, including pathological antibodies, pathological complement, irAE-associated cytokines (7), and the accumulated bilirubin and pseudo-neurotransmitters. However, to date, no established guidelines have explicitly recommended the specific application of PE and the time point for its implementation. In the present study, five of eight steroid-refractory patients (62.5%) responded to the PE treatment. The reported cases of successful PE in IMH patients mostly involved five sessions (31), but whether increased PE sessions can enhance the response rate remains uncertain. We observed a patient who initially received PE and subsequently experienced prolonged steroid treatment as well as subsequent infections; we also reported a case of an irAE-related pulmonary arterial hypertension patient whose pulmonary artery pressure elevated following PE (32). We believe that PE may also be a double-edged sword that clears both pathological factors related to side effects and factors that counteract these side effects in irAE progression. Early PE therapy may inadvertently remove protective antibodies or cytokines that help counteract irAEs. Based on this observation, we recommend performing a liver biopsy after confirming steroid resistance but prior to initiating PE therapy in order to avoid unnecessary interventions in patients with bile duct deficiency syndrome. In the future, we will try biological agents such as tocilizumab and infliximab, which have been proven to work in IMH treatment.

The infection rate among severe IMH patients receiving steroid treatment was 18.8%, including cytomegalovirus, novel coronavirus, and P.c. (**Supplementary Table S1**). Among the first 20 severe IMH patients, three cases of P.c. pneumonia were observed, resulting in one death. Under the condition of TBIL reduction upon steroid treatment, a prophylactic treatment of trimethoprim–sulfamethoxazole was performed for later severe IMH patients whose total steroid dosage reached the cutoff value of 1,656 mg. It did not induce excessive hepatotoxicity when patients’ TBIL was reduced to a median level of 166.9 μmol/L.

**Figure 2:**
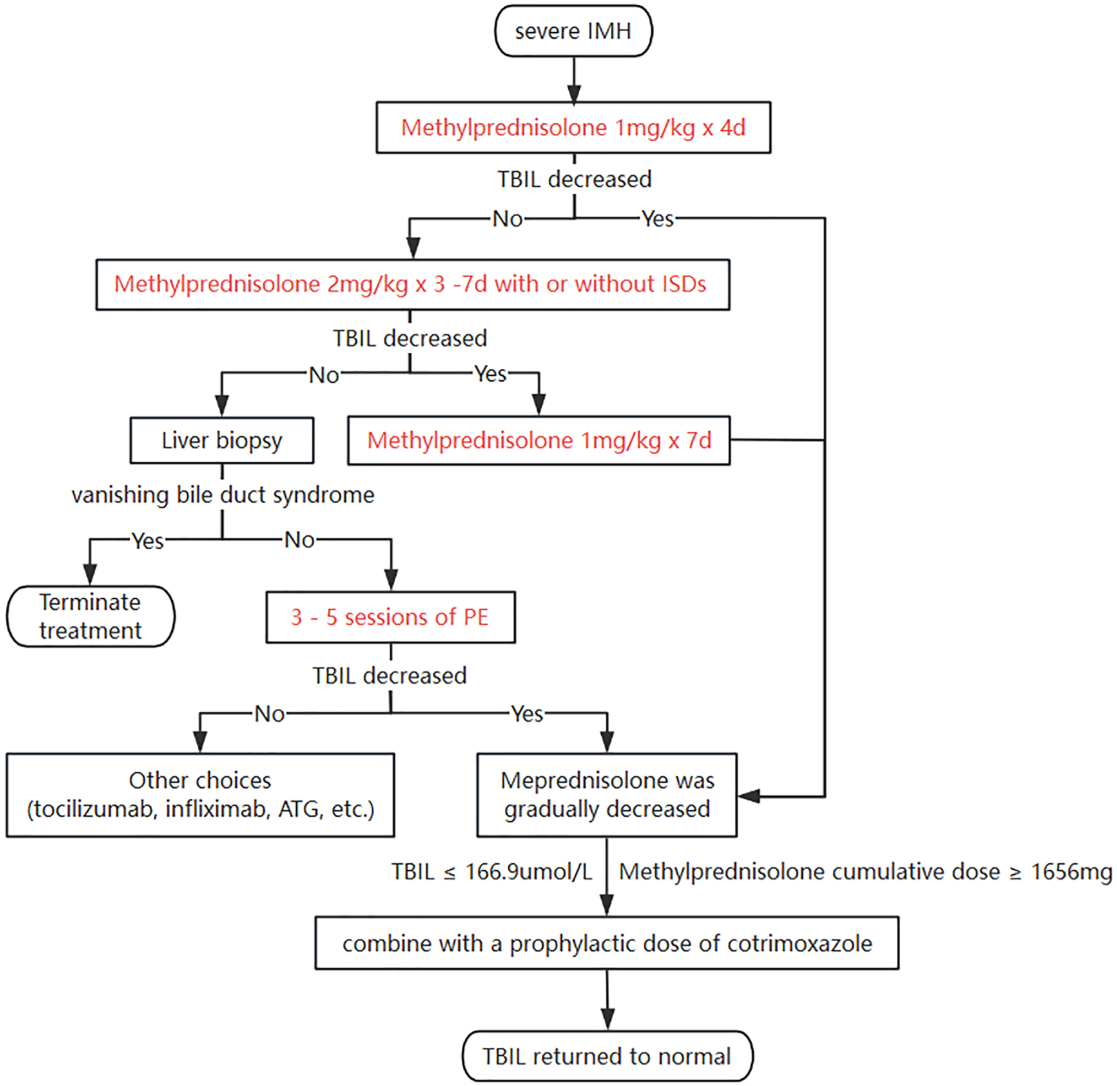
A roadmap for managing severe IMH. IMH, immune-mediated hepatotoxicity; TBIL, total bilirubin; ISDs, immunosuppressive drugs; PE, plasma exchange; ATG, antithymocyte globulin.

Based on the previous reports and our data, we provided a management algorithm for severe IMH (**Figure 2**). Methylprednisolone was administered at a dosage of 1 mg·kg^−1^·day^−1^ for 4 days, continuing with an increased dosage of 2 mg·kg^−1^·day^−1^ with or without immunosuppressive drugs (ISDs) for the non-responders to 11 days, and then a liver biopsy was performed for the remaining non-responders to exclude vanishing bile duct syndrome at that time point, followed by PE. During this process, when the steroid dosage reaches near the infection threshold and TBIL has dropped to 166.9 μmol/L or lower, cotrimoxazole should be used to prevent P.c. According to the proposed therapeutic algorithm for severe IMH, three out of 32 (9.4%) patients at our center experienced treatment failure resulting in death, which is lower than the reported mortality rate of 22% for severe IMH patients in other studies (8).

The present study had several limitations. First, it was a retrospective single-center study with a small sample size. Second, due to the limited sample size, we were unable to analyze potential correlations between factors associated with the occurrence of irAEs—such as family history of autoimmune diseases, prior episodes of other irAEs, use of proton pump inhibitors or metformin, and the development as well as treatment outcomes of IMH. These associations will be further explored in subsequent studies with larger sample sizes. Third, the overrepresentation of certain cancer types (e.g., lung, esophageal, and gastric cancers) in the cohort introduces a notable selection bias, although this variable was not included in the multivariate analysis. Given the limitation of sample size, this study inevitably carries the risk of confounding biases, particularly concerning the two clinical characteristics of TCM usage and ECOG PS scores. Fourth, due to the validity of the sample size, we were unable to conduct an internal validation of the ROC curve’s AUC of 0.923 to assess its stability. Fifth, there was an absence of histological analysis for all IMH patients to guide diagnosis and treatment. The necessity of liver biopsy in severe IMH cases remains controversial. According to the current NCCN guidelines for irAE management, the diagnosis of IMH is primarily one of exclusion. Liver biopsy should be conducted only when deemed absolutely necessary (on rare occasions) and not as a routine diagnostic measure (18). Furthermore, limited data exist regarding the histopathologic findings specific to IMH, and no pathognomonic features have been identified, thereby limiting the diagnostic utility of liver biopsy (33–35). Finally, given the invasive nature and associated risks of liver biopsy, it was selectively performed in this study only on patients with steroid resistance to guide further therapeutic strategies.

## Conclusion

We investigated the potential risk factors for severe IMH and provided a feasible treatment roadmap for its management. We hope that our findings can provide some assistance to clinical doctors in dealing with severe IMH.

## Data Availability

The original contributions presented in the study are included in the article/**Supplementary Material**. Further inquiries can be directed to the corresponding author.

## Supplementary material

Supplementary Figure 1 Changes in TBIL levels among patients with severe IMH. TBIL, total bilirubin; IMH, immune-mediated hepatotoxicity.
