# Supplementary figures and images for: Treatment experience in managing severe immune-mediated hepatotoxicity induced by immune checkpoint inhibitors
Source: Front Oncol. 2025 Oct 10;15:1657332. doi: 10.3389/fonc.2025.1657332 (PMC12549241; doi:10.3389/fonc.2025.1657332)

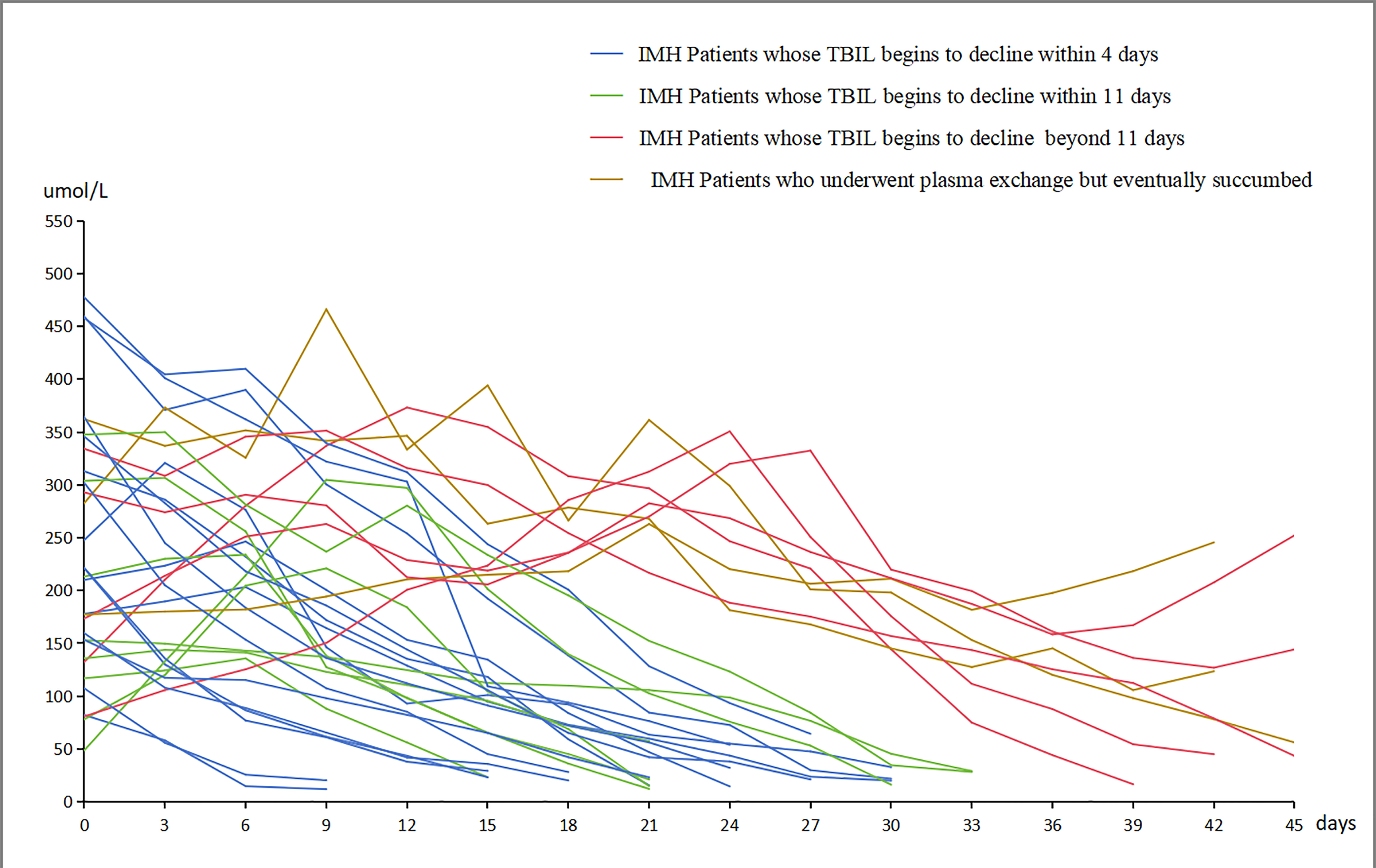

Supplement: Supplementary Figure 1 — Changes in TBIL levels among patients with severe IMH. TBIL, total bilirubin; IMH, immune-mediated hepatotoxicity. [file Image1.tif]
