# Supplementary material for: Treatment experience in managing severe immune-mediated hepatotoxicity induced by immune checkpoint inhibitors
Source: Front Oncol. 2025 Oct 10;15:1657332. doi: 10.3389/fonc.2025.1657332 (PMC12549241; doi:10.3389/fonc.2025.1657332)
Supplement: Supplementary file 2 [file Table1.docx]

**Table S1** Characteristics of patients who developed infections during steroid therapy in individuals with immune-mediated hepatotoxicity.

|  | Sex | Age | Primary cancer | ICIs | CTCAE grade | Time between immunotherapy and IMH  (days) | Types of infection | The dosage (mg)/duration of hormone (days) until the onset of infection | The TBIL upon initiation of treatment or prophylaxis with sulfamethoxazole (μmol/L) | Time from infection to resolution of IMH  (days) |
| --- | --- | --- | --- | --- | --- | --- | --- | --- | --- | --- |
| 1 | M | 60 | EC | Camrelizumab | G4 | 135 | P.c. | 840/14 | 92.6 | 28 |
| 2 | M | 68 | NSCLC | Camrelizumab | G4 | 45 | P.c. | 2300/40 | 32.8 | 12 |
| 3 | M | 59 | EC | Tislelizumab | G4 | 45 | P.c. | 1980/48 | 135.1 | Died of infection |
| 4 | F | 69 | SCLC | Adebrelimab | G4 | 300 | Novel coronavirus | 920/16 | 198.7 | 50 |
| 5 | M | 76 | NSCLC | Tislelizumab | G4 | 153 | CMV | 1260/20 | 235.2 | 56 |
| 6 | M | 71 | NSCLC | Tislelizumab | G4 | 76 | bacterial pneumonia | 2100/23 | 319.9 | 45 |

CMV, cytomegalovirus; CTCAE, Common Terminology Criteria for Adverse Events; EC, esophagus cancer; F, female; ICIs, immune checkpoint inhibitors; IMH, immune-mediated hepatotoxicity; M, male; NSCLC, non-small cell lung cancer; P.c., Pneumocystis carinii; SCLC, small cell lung cancer; TBIL, total bilirubin.
